# Supplementary material for: High-resolution neutron imaging of salt precipitation and water transport in zero-gap CO2 electrolysis
Source: Nat Commun. 2022 Oct 15;13:6099. doi: 10.1038/s41467-022-33694-y (PMC9569375; doi:10.1038/s41467-022-33694-y)
Supplement: Supplementary file 1 — Supplementary Information [file 41467_2022_33694_MOESM1_ESM.pdf]

Supplementary Information for

High-resolution neutron imaging of salt precipitation and water transport in zero-gap CO_2 electrolysis

Joey Disch[1,2], Luca Bohn[1,2], Susanne Koch[1,3], Michael Schulz[4], Yiyong Han[4], Alessandro Tengattini[5,6], Lukas Helfen[6], Matthias Breitwieser[1,3] and Severin Vierrath[1,2,3,*]

Correspondence to: Severin.Vierrath@imtek.uni-freiburg.de

[1] Electrochemical Energy Systems, IMTEK - Department of Microsystems Engineering, University of Freiburg, Georges-Koehler-Allee 103, 79110 Freiburg, Germany
[2] University of Freiburg, Institute and FIT – Freiburg Center for Interactive Materials and Bioinspired Technologies, Georges-Köhler-Allee 105, 79110 Freiburg
[3] Hahn-Schickard, Georges-Koehler-Allee 103, 79110 Freiburg, Germany
[4] Heinz Maier-Leibnitz Zentrum (MLZ), Technische Universität München, Garching, Germany
[5] Grenoble INP, CNRS, 3SR, Univ. Grenoble Alpes, 38000, Grenoble, France
[6] Institute Laue-Langevin, 71 avenue des Martyrs - CS 20156, 38042 Grenoble, France
*corresponding author

Contents

Supplementary Fig. 1 Cell fixture and neutron detector/cell alignment. 2
Supplementary Fig. 2 Schematic drawing of the electrolysis test setup. 2
Supplementary Fig. 3 Electrochemical measurements at 50 °C cell temperature. 3
Supplementary Fig. 4 Selected regions for cathode GDL (left), membrane (middle) and anode channel (right) vertical profiles of Figure 4. 4
Supplementary Fig. 5 Selected regions for channel and land area comparison in Figure 5. 4
Supplementary Table 1 Neutron transmission rates for different substances and materials at a wavelength of 3 Å. 5
Supplementary Fig. 6 Calculated neutron transmission for different compounds and sample thicknesses. 5
Supplementary Fig. 7 Scanning electron micrographs and energy dispersive X-ray spectroscopies of the cathode electrode cross section after cell operation. 6
Supplementary Fig. 8 Raman spectra of pure potassium bicarbonate, potassium carbonate, and of the cathode catalyst layer before and after cell operation. 7

Experimental setup

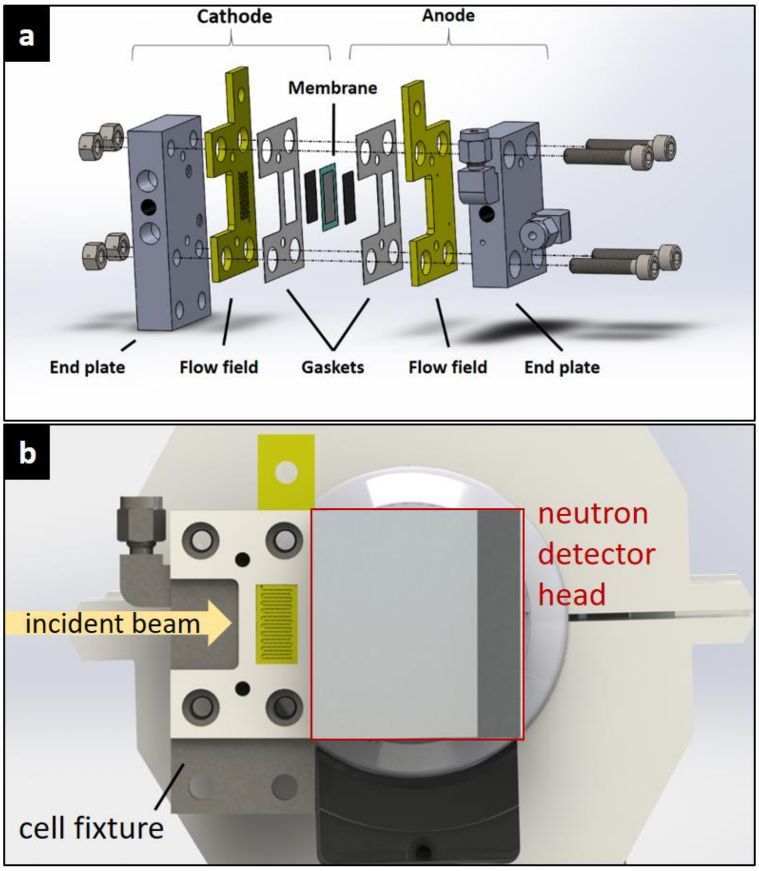

Supplementary Fig. 1 Cell fixture and neutron detector/cell alignment. a an explosion view of the custom-built electrolysis cell (End plates: Stainless steel, flow fields: Au coated Ti grade 2, gaskets: PTFE). **b** a rendering of the neutron detector and of the cell fixture cross section in alignment to the incident neutron beam. The scintillator sits in the neutron detector head close to the cell fixture. The emitted scintillation light is reflected to the detector, which is orientated perpendicular to the incident neutron beam. Two heating cartridges inserted in the endplates (two holes going in from the front) can heat up the cell.

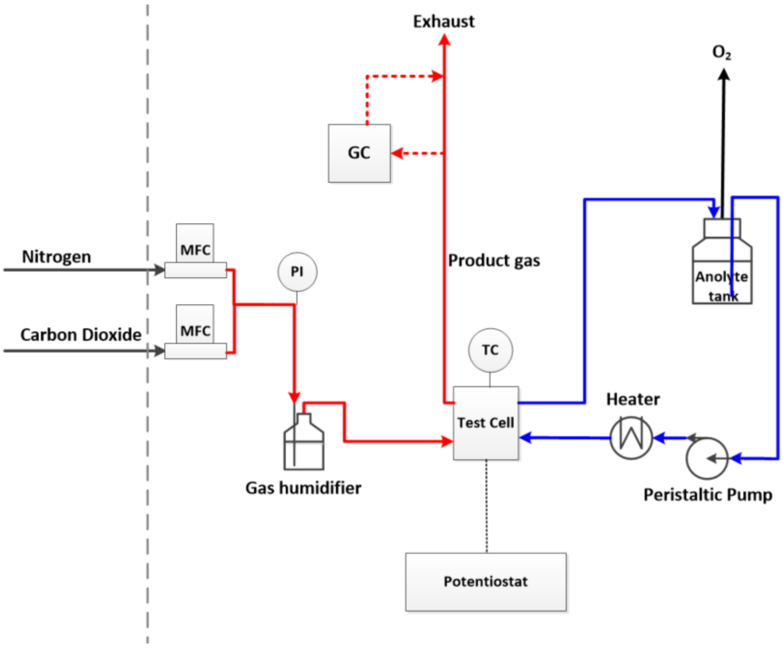

Supplementary Fig. 2 Schematic drawing of the electrolysis test setup (MFC: Mass flow controller, PI: Pressure sensor, TC: Temperature controller, GC: Gas chromatograph).

Electrochemical measurements at 50 °C cell temperature

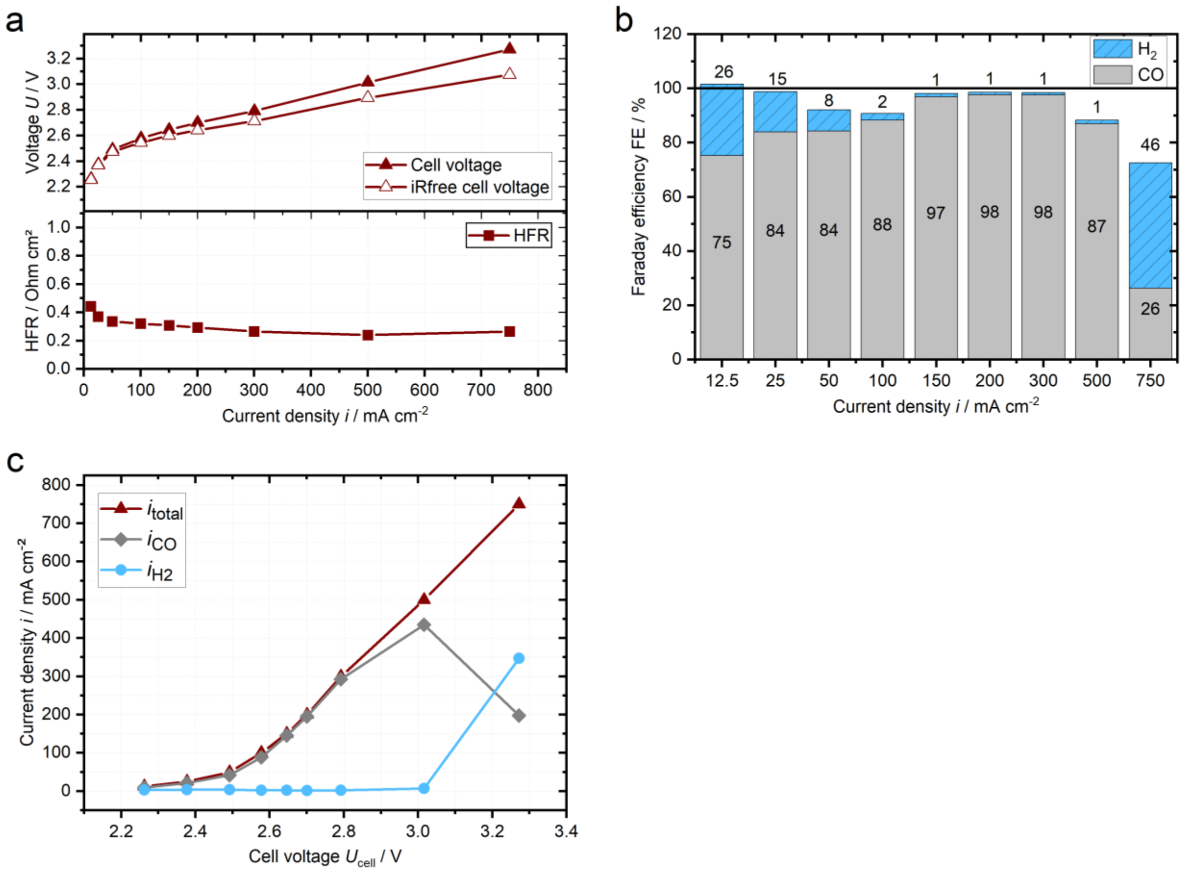

Supplementary Fig. 3 Electrochemical measurements at 50 °C cell temperature. **a** VI-Curve, **b** Faraday efficiencies and **c** partial current densities of the identical cell (cell 2) at 50 °C cell temperature (0.1 M KOH anolyte, 10 min current steps). Measured additionally after completing the replication measurements.

Neutron transmission

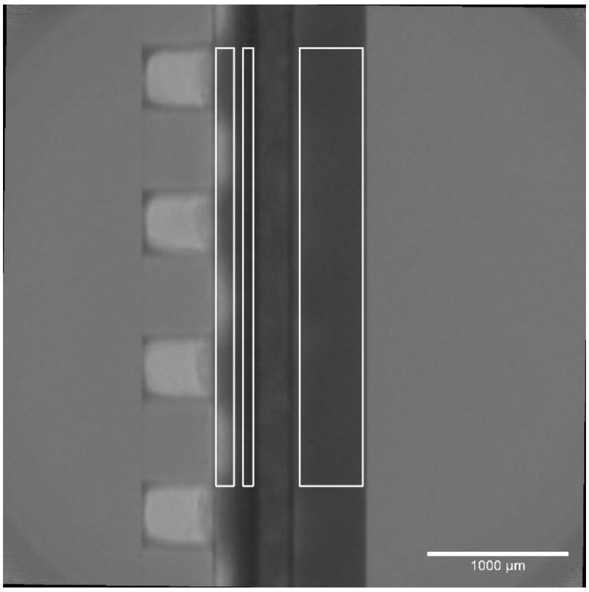

Supplementary Fig. 4 Selected regions for cathode GDL (left), membrane (middle) and anode channel (right) vertical profiles of Figure 4.

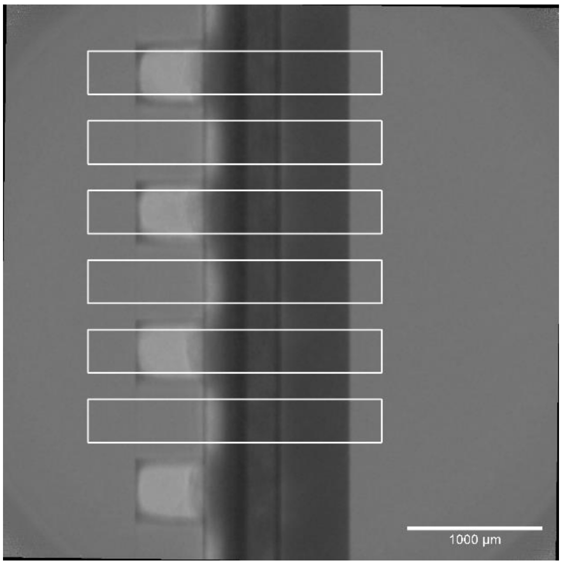

Supplementary Fig. 5 Selected regions for channel and land area comparison in Figure 5.

Supplementary Table 1 Neutron transmission rates for different substances and materials at a wavelength of 3 Å. *calculated with the online calculator from "NIST Center for Neutron Research" [https://www.ncnr.nist.gov/resources/activation/, Date: 02.2022)]. The width of the active cell area in beam direction is 1 cm.

	thickness / cm	density / kg/L	neutron transmission*
H_2O	1	1.00000	0.0%
$KHCO_3$	1	2.17000	30.3%
K_2CO_3	1	2.43000	96.0%
H_2	1	0.00009	99.6%
CO_2	1	0.00198	100.0%
CO	1	0.00125	100.0%
HCOOH	1	1.22000	6.1%
KCOOH	1	1.91000	28.8%
C	1	2.26000	100.0%
Au	1	19.32000	3.9%
Ti	1	4.50000	70.0%
H_2O vapor	1	0.00059	99.7%

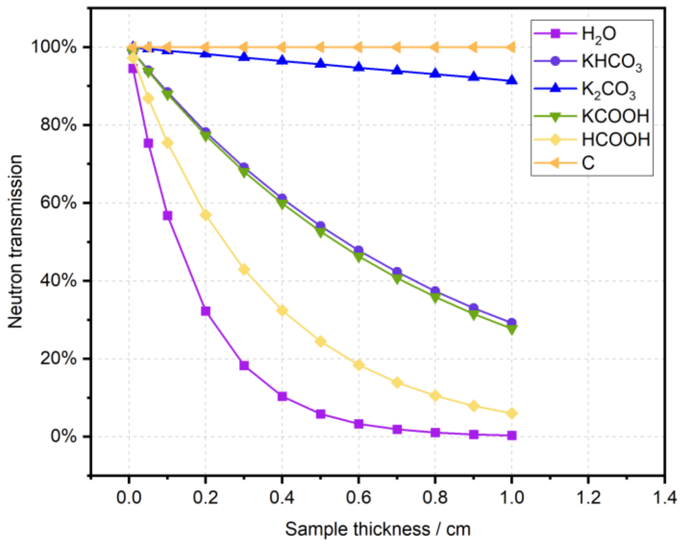

Supplementary Fig. 6 Calculated neutron transmission for different compounds and sample thicknesses calculated with the online calculator from "NIST Center for Neutron Research" [https://www.ncnr.nist.gov/resources/activation/, Date: 02.2022]. The wavelength was set to 3 Å.

Ex situ characterization

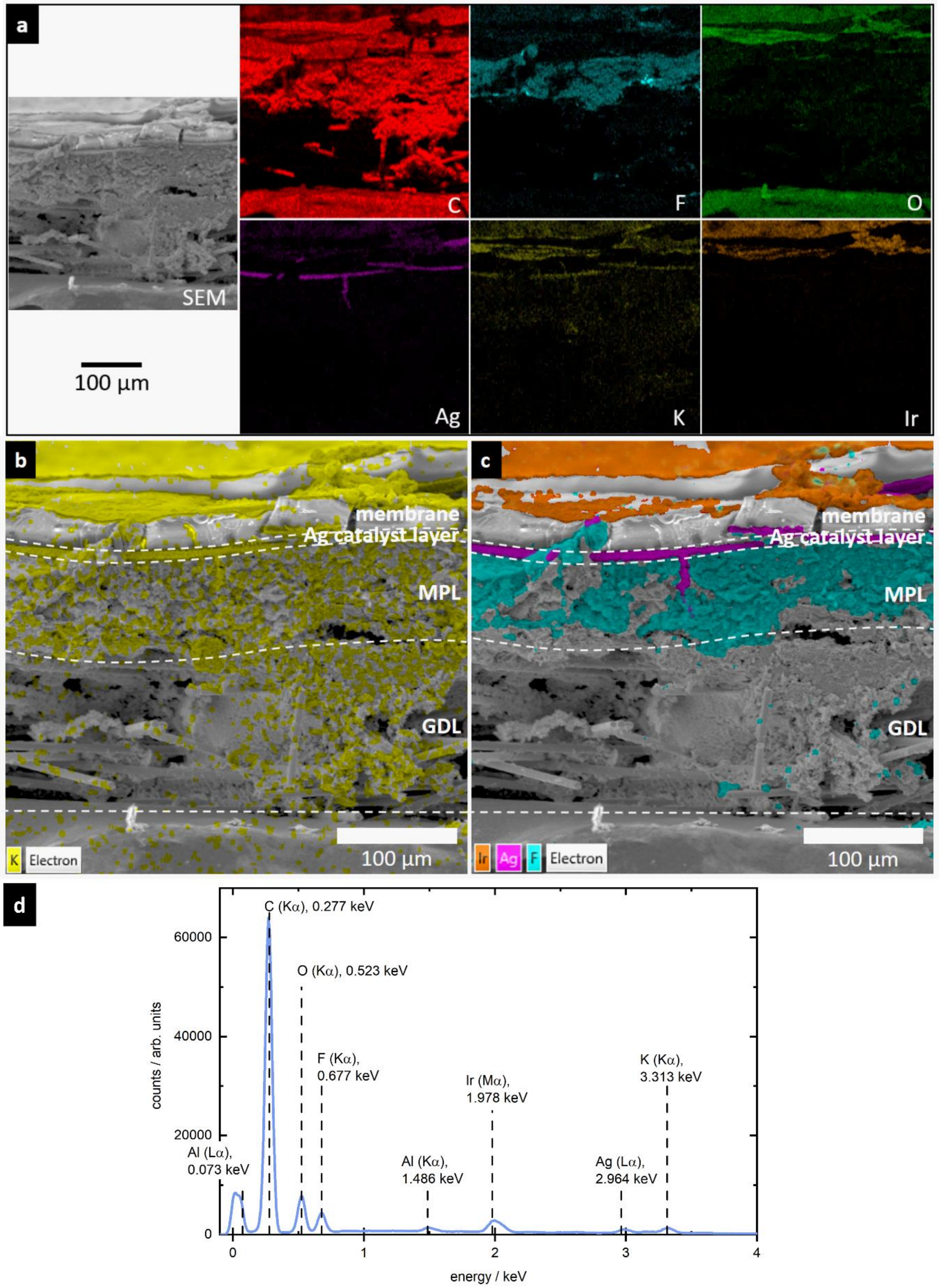

Supplementary Fig. 7 Scanning electron micrographs and energy dispersive X-ray spectroscopies of the cathode electrode cross section after cell operation. a electron micrograph and elemental maps of carbon, fluorine, oxygen, silver, potassium and iridium. **b** a layered image of the electron micrograph and the elemental map of potassium, **c** an electron micrograph layered with the elemental maps of iridium, silver and fluorine, identifying the catalyst layers and the hydrophobic microporous layer and **d** spectrum obtained from the energy dispersive X-ray spectroscopy. The cross section was prepared by cryo-cutting.

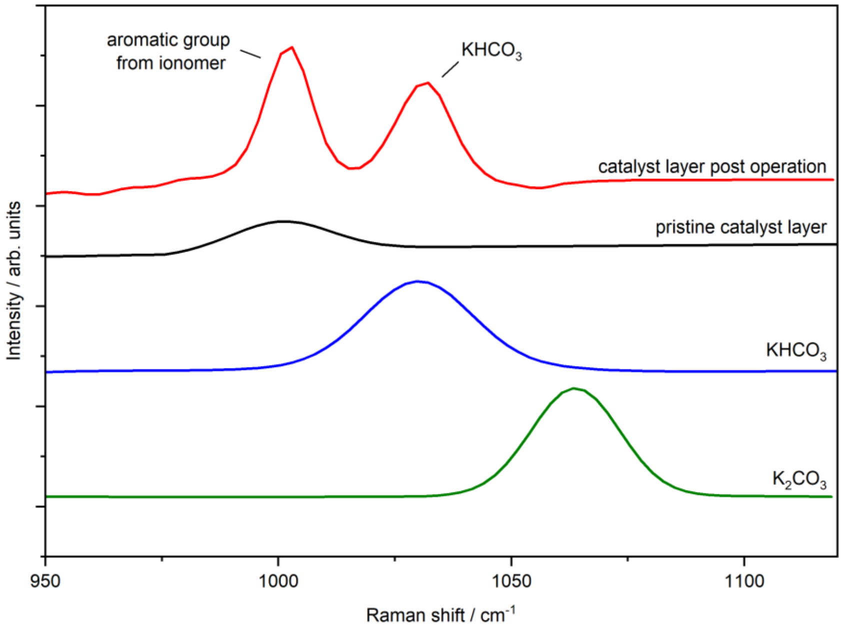

Supplementary Fig. 8 Raman spectra of pure potassium bicarbonate, potassium carbonate, and of the cathode catalyst layer before and after cell operation. The post operation spectra were recorded at different positions of the cathode GDE catalyst layer surface after carefully removing the membrane. The aromatic group signal results from the Sustainion cathode catalyst binder[1,2]. Raman spectra were obtained using a WITec alpha 300 confocal Raman microscope with a 532 nm laser operated at 10 ± 1 mW as the excitation source. Average spectra of the samples were produced by averaging five single spectra from each respective sample. All single spectra were integrated for 0.5 s and accumulated ten times. Background subtraction and fitting was done using WITec project.

References

1. Kutz, R. B. *et al.* Sustainion Imidazolium-Functionalized Polymers for Carbon Dioxide Electrolysis. *Energy Technol.* **5,** 929–936; 10.1002/ente.201600636 (2017).

2. Nwabara, U. O. *et al.* Binder-Focused Approaches to Improve the Stability of Cathodes for CO 2 Electroreduction. *ACS Appl. Energy Mater.* **4,** 5175–5186; 10.1021/acsaem.1c00715 (2021).
